# Survival improvements of marine mammals in zoological institutions mirror historical advances in human longevity

**DOI:** 10.1098/rspb.2023.1895

**Published:** 2023-10-18

**Authors:** Morgane Tidière, Fernando Colchero, Johanna Staerk, Michael J. Adkesson, Ditte H. Andersen, Lucie Bland, Martin Böye, Sabrina Brando, Isabella Clegg, Sarah Cubaynes, Amy Cutting, Danny De Man, Andrew E. Derocher, Candice Dorsey, William Elgar, Eric Gaglione, Kirstin Anderson Hansen, Allison Jungheim, José Kok, Gail Laule, Agustín Lopez Goya, Lance Miller, Tania Monreal-Pawlowsky, Katelyn Mucha, Megan A. Owen, Stephen D. Petersen, Nicholas Pilfold, Douglas Richardson, Evan S. Richardson, Devon Sabo, Nobutaka Sato, Wynona Shellabarger, Cecilie R. Skovlund, Kanako Tomisawa, Sandra E. Trautwein, William Van Bonn, Cornelis Van Elk, Lorenzo Von Fersen, Magnus Wahlberg, Peijun Zhang, Xianfeng Zhang, Dalia A. Conde

**Affiliations:** ^1^ Interdisciplinary Centre on Population Dynamics (CPop), University of Southern Denmark, Odense, Denmark; ^2^ Department of Mathematics and Computer Science, University of Southern Denmark, Odense, Denmark; ^3^ Department of Biology, University of Southern Denmark, Campusvej 55, 5230 Odense, Denmark; ^4^ Conservation and Science Department, Species360, 7900 International Drive, Suite 300, Minneapolis, MN 55425, USA; ^5^ Department of Primate Behavior and Evolution, Max Planck Institute for Evolutionary Anthropology, Deutscher Pl. 6, 04103 Leipzig, Germany; ^6^ Chicago Zoological Society, Brookfield Zoo, Brookfield, IL, USA; ^7^ Eureka Publishing, Thornbury, Australia; ^8^ Centre de Recherche et d'Etude pour l'Animal Sauvage, Planète Sauvage, 44710 Port Saint Pere, France; ^9^ AnimalConcepts, PO Box 378, 03725 Teulada, Alicante, Spain; ^10^ Animal Welfare Expertise, The Knoll, Woodlands, Combe Martin, EX34 0ATLittleton Manor, Winchester SO22 6QU, UK; ^11^ CEFE, Univ Montpellier, CNRS, EPHE-PSL University, IRD, Montpellier, France; ^12^ Polar Bear International, PO Box 3008, Bozeman, MT, USA; ^13^ European Association of Zoos and Aquaria (EAZA), Plantage Middelaan 45, 1018-DC Amsterdam, The Netherlands; ^14^ Department of Biological Sciences, University of Alberta; Edmonton, Alberta, Canada T6G 2E9; ^15^ Association of Zoos and Aquariums, 8403 Colesville Road Ste 710, Silver Spring, MD 20910, USA; ^16^ Zoo Miami, 12400 SW 152 Street, Miami, FL 33177, USA; ^17^ Georgia Aquarium, 225 Baker Street, Atlanta, GA 30313, USA; ^18^ Marine Biological Research Center, University of Southern Denmark, Hindsholmvej 11, 5300 Kerteminde, Denmark; ^19^ Como Park Zoo and Conservatory, 1225 Estabrook Dr., Saint Paul, MN 55103, USA; ^20^ Ouwehands Zoo, Grebbeweg 111, 3911 AV Rhenen, The Netherlands; ^21^ Mandai Wildlife Group, 80 Mandai Lake Road, Singapore 729826; ^22^ Zoo Aquarium de Madrid, Casa de Campo s/n, 28011 Madrid, Spain; ^23^ International Zoo Veterinary Group, Keighley, UK; ^24^ San Diego Zoo Wildlife Alliance, 15600 San Pasqual Valley Rd., Escondido, CA, USA; ^25^ Assiniboine Park Zoo, Winnipeg, Manitoba, Canada; ^26^ Zoological Consultancy Ltd, Columba Cottage, Mill Rd, Kingussie PH21 1LF, UK; ^27^ EAZA Polar Bear EEP, Amsterdam, Netherlands; ^28^ Environment and Climate Change Canada, Unit 150–234 Donald Street, Winnipeg, Manitoba R3C 1M8, Canada; ^29^ Columbus Zoo and Aquarium, 4850 W. Powell Road, PO Box 400, Powell, OH 43065-0400, USA; ^30^ Asahiyama Zoological Park, Kuranuma, Higasiasahikawacho, Asahikawa city, Japan; ^31^ Detroit Zoological Society, 8450 W. 10 Mile Rd, Royal Oak, MI 48067, USA; ^32^ Conservation, Copenhagen Zoo, Roskildevej 38, 2000 Frederiksberg, Denmark; ^33^ Section of Animal Welfare and Disease Control, Department of Veterinary and Animal Sciences, University of Copenhagen, Grønnegårdsvej 8, 1870 Frederiksberg, Denmark; ^34^ Omuta City Zoo, 163 Showa-machi, Omuta, Fukuoka 836-0871, Japan; ^35^ A. Watson Armour III, Center for Animal Health and Welfare, Animal Care and Science Division, John G. Shedd Aquarium, Chicago, IL 60605, USA; ^36^ Independent practitioner, Arendsweg 98, Enschede 7544RM, The Netherlands; ^37^ Nuremberg Zoo, Am Tiergarten 30, 90480 Nuremberg, Germany; ^38^ Mammal and Marine Bioacoustics Laboratory Institute of Deep-sea Science and Engineering, Chinese Academy of Sciences, Sanya 572000, People's Republic of China; ^39^ Institute of Hydrobiology, Chinese Academy of Sciences, Wuhan 430072, People's Republic of China

**Keywords:** adult mortality, first-year mortality, life expectancy, lifespan equality, population welfare

## Abstract

An intense public debate has fuelled governmental bans on marine mammals held in zoological institutions. The debate rests on the assumption that survival in zoological institutions has been and remains lower than in the wild, albeit the scientific evidence in support of this notion is equivocal. Here, we used statistical methods previously applied to assess historical improvements in human lifespan and data on 8864 individuals of four marine mammal species (harbour seal, *Phoca vitulina*; California sea lion, *Zalophus californianus*; polar bear, *Ursus maritimus*; common bottlenose dolphin, *Tursiops truncatus*) held in zoos from 1829 to 2020. We found that life expectancy increased up to 3.40 times, and first-year mortality declined up to 31%, during the last century in zoos. Moreover, the life expectancy of animals in zoos is currently 1.65–3.55 times longer than their wild counterparts. Like humans, these improvements have occurred concurrently with advances in management practices, crucial for population welfare. Science-based decisions will help effective legislative changes and ensure better implementation of animal care.

## 1. Introduction

In recent years, a sustained debate around animal welfare in zoological institutions (i.e. zoos, aquariums, rescue centres and wildlife sanctuaries) has fuelled government bans despite poor scientific evidence [1,2]. In 2017, SeaWorld USA announced the end of their orca (*Orcinus orca*) breeding programme due to pressure from animal rights groups following the release of the film *Blackfish*. Countries such as Canada and Switzerland have banned breeding certain marine mammal species (mostly whales and Delphinidae species), while other jurisdictions debate whether to follow suit. Yet, large-scale scientific evidence on the welfare of marine mammal populations in zoological institutions and studies on survival compared with wild counterparts is only beginning to emerge [3–8].

At the population level, a linear increase in two demographic measures, namely life expectancy and lifespan equality, has provided a robust welfare indicator for human societies [9]. Life expectancy is defined as the average lifespan in a population, while lifespan equality measures the concentration of deaths at older ages relative to life expectancy. Research in humans [10] and other animals [11,12] has shown that improvement in welfare is associated with longer lifespans through both direct (e.g. medical care) and indirect mechanisms (e.g. cumulative effects of positive physical and mental welfare). In association with increased life expectancy, lifespan equality has risen in industrial human societies, where deaths are concentrated at older ages compared to pre-industrial and hunter–gatherer populations [9]. Non-human primates living in zoological institutions also have higher life expectancy and lifespan equality than their wild counterparts, potentially reflecting the effect of managed care and environments on their demography [12]. For now, it remains unknown whether this linear increase in life expectancy and lifespan equality occurs in non-primate taxonomic groups, and whether changes in these two metrics can be related to changes in zoological management and practices that promote population welfare.

To assess long-term changes (1829–2020) in population welfare for marine mammals living in zoological institutions, we estimated age-specific mortality for males and females of the harbour seal (*Phoca vitulina*, *n* = 1907), California sea lion (*Zalophus californianus*, *n* = 3940), polar bear (*Ursus maritimus*, *n* = 2025) and common bottlenose dolphin (*Tursiops truncatus*, *n* = 992). These 8864 individuals represent 63.4% of all marine mammals recorded in the global Species360 Zoological Information Management System (ZIMS) [13] since 1829 (electronic supplementary material, figure S1). Based on species prevalence in zoological institutions across time, and to ensure adequate sample sizes for modelling (see Methods), we defined four periods for the California sea lion and polar bear (pre-1975, 1975–1989, 1990–2004 and 2005–2020) and three periods for the harbour seal and common bottlenose dolphin (pre-1990, 1990–2004 and 2005–2020). To ensure comparability with wild populations (where first-year mortality is difficult to estimate accurately), we derived the remaining life expectancy and lifespan equality for individuals in zoological institutions reaching at least 1 year of age (hereafter, life expectancy and lifespan equality). In addition, based on published sex-specific survival data [14–20], we estimated sex-specific life expectancy and lifespan equality from age one for at least one wild population of each species. Finally, to ensure that our results were not only related to high mortality early in life, we repeated our analyses from birth and age at sexual maturity.

## 2. Methods

### (a) Species data

Records were obtained from the ZIMS managed by Species360, a non-profit organization with over 1300 current members all around the world, including zoos, aquariums, rescue centres and wildlife sanctuaries [13]. Records included information on individuals living in zoological institutions from the early 1800s to 1 January 2021. The harbour seal, California sea lion, polar bear and common bottlenose dolphin were retained for the study because the database contained at least 100 individuals per sex for each period and species, to ensure unbiased mortality estimates and minimize uncertainty (as suggested in [21]). These four species are currently the most represented marine mammals in zoological institutions (representing 63.4% of marine mammals in ZIMS; electronic supplementary material, figure S1).

We developed survival analyses from birth, age one and age at sexual maturity. To avoid possible data entry errors for each species, we excluded records with unknown sex from the dataset, as well as the 1% longest-lived individuals (electronic supplementary material, figure S2). Individuals whose sex was not recorded accounted for 14.4% of the records, most of which died in their first year of life (electronic supplementary material, figure S2). We obtained a final dataset of 1907 harbour seals, 3941 California sea lions, 2025 polar bears and 992 common bottlenose dolphins. We analysed the data into three time periods of at least 15 years, for the harbour seal and common bottlenose dolphin (pre-1990, 1990–2004 and 2005–2020). For California sea lion and polar bear, we defined four periods (pre-1975, 1975–1989, 1990–2004 and 2005–2020). Final sample sizes for each species, sex and period are available in electronic supplementary material (table S1).

For comparison, we obtained sex-specific and age- or stage-specific survival data for at least one wild population per species. For the harbour seal, we extracted the published estimated age structure of 2145 individuals from the Skagerrak, the Kattegat and the Baltic Seas (before the 2002 epidemic of Phocine Distemper Virus) [14], and stage-specific survival for 347 individuals from the Tugidak Island (Alaska) monitored between 2000 and 2007 [18]. For the California sea lion, we obtained stage-specific survival probabilities for 196 individuals monitored in the Gulf of California from 1980 to 2006 [17], and life tables from 11 298 individuals from the San Miguel Island (California) from 1987 to 2015 [19]. For the polar bear, we obtained stage-specific survival probabilities from 1963 individuals (across 3306 captures) in western Hudson Bay, Canada, from 1984 to 2004 [15]. Finally, for the common bottlenose dolphin, we obtained sex-specific life tables from 220 individuals monitored in the Indian river lagoon system (Florida) from 1978 to 1997 [16] and from 111 individuals from a population living in the north-central Gulf of Mexico monitored between 1986 and 2003 [20].

### (b) Data analysis

#### (i) Survival analysis

To draw inferences on age-specific mortality and survival when individual ages are missing, we used the Bayesian survival trajectory analysis (BaSTA) [21,22] in R [23]. We modified the R package BaSTA [22], originally designed for capture-mark-recapture data, to analyse ZIMS census data. As with most survival analysis methods, BaSTA makes inferences on age-specific survival from records that may include left-truncation (i.e. individuals that enter the study after birth) and right-censoring (i.e. individuals that drop out of the study before death) (see below for a description of the likelihood function). Moreover, BaSTA allows the inclusion of individuals for which the time of birth is uncertain, expanding the pool of available data for analysis.

To define mortality patterns, we fitted a Siler mortality model [24], the most parsimonious model for the shape of age-mortality changes in most mammal species with three stages of maturity (i.e. juvenile, maturity and senescence) [25]. The Siler model describes the hazard rate or mortality function as a convex function of age *x* given by

2.1 $$\mu (x)=\exp(a_{0}-a_{1}x)+c+\exp(b_{0}+b_{1}x),$$

where *a*_0_, *b*_0_ ∈ (−∞, +∞) and *a*_1_, *c*, *b*_1_ > 0 are mortality parameters to be estimated. This function includes an initial decline in juvenile mortality, given by the first exponential term in equation (2.1). The second part of the equation is a Gompertz model assuming mortality increases exponentially with age after sexual maturity. Parameters *a*_0_ and *a*_1_ control the initial level and the rate of decline in juvenile mortality, respectively. Parameter *c* accounts for age-independent mortality, and parameters *b*_0_ and *b*_1_ control the increase in adult and senescent mortalities [26–28]. The cumulative hazard is given by

2.2 $$U(x)=\int_{0}^{x}\mu (t)dt,$$

and the survival function is calculated as

2.3 S(x)=exp[−U(x)],

which is the complement of the cumulative distribution function of ages at death, *F*(*x*) = 1–*S*(*x*), and the probability density function of ages at death is given by

2.4 f(x)=μ(x)S(x).

The likelihood function is therefore

2.5 $$L(x,x_{t}|\theta )=\{\begin{array}{cc} \frac{\;f(x)}{S(x_{t})} & \mathrm{if}\;\mathrm{uncensored}\\ \frac{S(x)}{S(x_{t})} & \mathrm{if}\;\mathrm{censored} \end{array}$$

where *x_t_* < *x* is the age at left-truncation (*x_t_* = 0 for individuals born during the study period), and where ***θ***^T^ = [*a*_0_, *a*_1_, *c*, *b*_0_, *b*_1_] is the vector of mortality parameters to be estimated. BaSTA uses Markov chain Monte Carlo with Metropolis–Hasting sampling for the unknown mortality parameters and times of birth [29,30]. We ran eight parallel chains of 50 000 iterations each, with a burn-in of 10 000 and thinning each 20 iterations. From the resulting parallel chains, we calculated measures of convergence based on the potential scale reduction proposed by Gelman *et al*. [31]. We then reconstructed the posterior densities of the estimated parameters and other additional measures (i.e. life expectancy and lifespan equality), from which 95% credible intervals can be obtained.

We then extracted the life tables from the BaSTA outputs for each period, sex and species (electronic supplementary material, appendix S1). These are constructed from the raw data for individuals with known time of birth and based on the estimated average time of birth for those with uncertain birth date. BaSTA uses a product limit estimator to construct non-parametric survival curves [32], and then reconstructs the life tables over discrete age intervals.

Given that captive-born individuals can have survival advantages over wild-born ones (e.g. [5]), we performed additional Bayesian survival trajectory analyses, including the provenance as a proportional hazard (i.e. wild-born, captive-born, not reported) per sex and species.

#### (ii) Life expectancy, lifespan equality and first-year mortality for populations living in zoological institutions

Patterns of age-specific mortality and longevity can be described by means of summary statistics such as the life expectancy (theoretical average age at death), and by measures that relate to the relative variation in the length of life (i.e. lifespan equality). Remaining life expectancy (life expectancy hereafter) from age *x* is given by

2.7 $$e_{x}=\frac{\int_{x}^{\infty }S(t)dt}{S(x)}.$$

To obtain the lifespan equality values, we first calculated lifespan inequality [33,34] from age *x* as

2.8 $$H_{x}=\frac{\int_{x}^{\infty }S(t)/S(x)[U(t)-U(x)]dt}{e_{x}}.$$

Thus, lifespan inequality provides a weighted average of the accumulation of deaths, weighted by cumulative survival. Therefore, *H_x_* increases as the ages at death become more widespread, and diminishes as they become more concentrated, particularly at older ages. We then calculated lifespan equality from age *x* as

2.9 $$\varepsilon_{x}=-\ln H_{x}\;.$$

Lifespan equality is, therefore, a dimensionless measure of the shape of the distribution of ages at death in a population after age *x*. Given that it is the log of the inverse of *H_x_*, lifespan equality measures the concentration of deaths at older ages; the higher the concentration at older ages, the higher the lifespan equality.

To account for potential issues of undetectability of early mortalities, particularly from the wild populations, and to ensure changes in life expectancy and lifespan equality were not solely related to changes in first-year mortality in zoological institutions, we calculated both metrics from birth, from age one and from age at sexual maturity [35].

In addition, to evaluate the changes in first-year mortality, we calculated the posterior densities of the mortality probabilities in the first year of age from the estimated Siler parameters as *q*_1_ = 1 – *S*(1), for females and males and all periods.

We then used Kullback–Leibler (K-L) discrepancies [36] to quantify the differences in life expectancy, lifespan equality and first-year mortality between the latest period (2005–2020) and those from the previous periods. The K-L discrepancies measure the amount of information lost if we predict, for instance, the life expectancy in the last period from the posterior density of any of the previous periods. Since K-L discrepancies are bound in the interval [0,∞), we used a calibration based on McCulloch [37] that limits them to the interval [0,1], improving interpretability. Here, a value of 0 implies no loss of information (i.e. that both densities are equal), and a value of 1 implies a complete loss of information (i.e. that both densities have no overlap).

#### (iii) Life expectancy and lifespan equality for wild populations

We used published survival data for wild populations from life tables or stage-specific survival probabilities or by digitizing cumulative survival plots. To make the estimates of life expectancy and lifespan equality comparable between the wild and ZIMS populations, we used least squares to fit Siler models to the age-dependent cumulative survival when available. For populations with stage-specific survival, we modified the least-squares algorithm by calculating the stage-specific survival probabilities from the Siler model as the weighted average of the age-specific survival probabilities, weighted by the cumulative survival given by

2.10 $$p_{x,x+n}=\frac{\sum_{i=x}^{x+n}p_{i}l_{i}}{\sum_{i=x}^{x+n}l_{i}},$$

where *p_x,x+n_* is the stage-specific survival probability in the age interval [*x*, *x* + *n*), *p_x_* is the age-specific survival probability where *p_x_* = *l_x_*/*l_x_*_−1_, and *l_x_* is the lifetable (discrete ages) cumulative survival probability calculated simply as *l_x_*=*S*(*x*) from the Siler model. In addition, we included the age at which the Siler mortality function reached a minimum value as a proxy for the age at maturity. The least-squares algorithm calculated the square differences between the stage-specific survival probabilities from the Siler model and those of the wild populations, as well as the square differences of the age at maturity from the wild population and the age at minimum mortality from the Siler model. By reconstructing the Siler mortalities for the wild populations, we obtained more accurate estimates, particularly of lifespan equality for those that only had stage data. We then verified that the Siler mortality estimates produced sensible values by comparing the resulting life expectancies to those approximated from the raw life table survival, calculated as

2.11 $$e_{x}\approx \;\frac{\sum_{i=x}^{\omega }l_{i}}{l_{x}},$$

where *ω* is the maximum age in the population.

Note that, since the data available from the wild populations were aggregated as survival estimates without measures of uncertainty, we reported their life expectancy and lifespan equality as point estimates. Nonetheless, we measured the quantiles of the wild estimates on the posterior densities of life expectancy and lifespan to measure the difference between wild and ZIMS estimates.

## 3. Results

**Figure 1.**
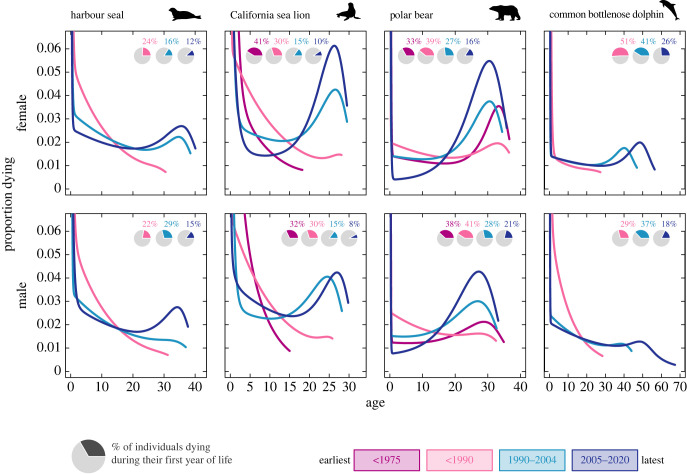
Distribution of ages at death (i.e. proportion of individuals dying at each age) by period for both sexes of zoo-held harbour seal (*Phoca vitulina*), California sea lion (*Zalophus californianus*), polar bear (*Ursus maritimus*) and common bottlenose dolphin (*Tursiops truncatus*) obtained from the output of the Bayesian survival trajectory analyses. Inset pie charts show the percentage of first-year mortality. See electronic supplementary material, table S1 for sample sizes. Silhouette images extracted from Phylopic.org (License CC0 1.0 Universal Public Domain Dedication; credits: *P. vitulina*: Tracy Heath, 29 June 2013; *Z. californianus*: Margot Michaud, 4 April 2021; *U. maritimus*: Margot Michaud, 4 April 2021; *T. truncatus*: Steven Traver, 22 Feb 2012).

Overall, the first-year mortality probability of individuals in zoological institutions decreased with time, from 22–51% in pre-1990 to 8–26% in 2005–2020 (figure 1; electronic supplementary material, figure S3), suggesting improvements in reproductive and juvenile care. Note that improved record keeping practices in recent years by which reporting of early deaths has increased means that the improvements we report here might be even more pronounced. Furthermore, in the more recent period, the distribution of ages at death showed a marked concentration at older ages for all species (figure 1). Pronounced old-age mortality modes were particularly evident for female California sea lions and polar bears. By contrast, the lower concentration of deaths at older ages for common bottlenose dolphins suggested that further improvements in longevity are possible.

Our results showed that the provenance did not affect the survival estimates in zoological institutions of three of the four species (electronic supplementary material, table S2). Only for common bottlenose dolphins, did we find that wild-born individuals had overall a lower survival than individuals born under human care. However, due to the negligible effect of provenance on the other three species and because most common bottlenose dolphins were wild born before 1990 (electronic supplementary material, figure S4), and therefore provenance being an important driver of the observed mortality only for the first period, we did not include it as a predictor in any subsequent analysis.

**Figure 2.**
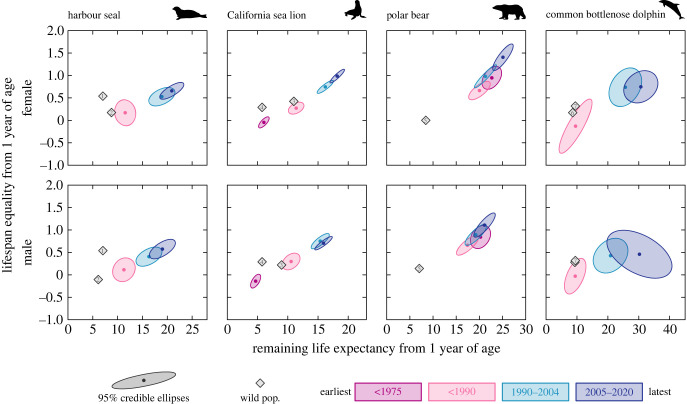
Life expectancy and lifespan equality from 1 year of age by period for both sexes of zoo-held harbour seal (*Phoca vitulina*), California sea lion (*Zalophus californianus*), polar bear (*Ursus maritimus*) and common bottlenose dolphin (*Tursiops truncatus*). For comparison, sex-specific values from wild populations are plotted (note: credible intervals could not be calculated for wild populations): 1. Härkönen *et al*. [14], 2. Hastings *et al*. [18], 3. Hernández-Camacho *et al*. [17], 4. Delong *et al*. [19], 5. Lunn *et al*. [15], 6. Stolen & Barlow [16], 7. Mattson *et al*. [20]. See electronic supplementary material, table S1. Silhouette images extracted from Phylopic.org (License CC0 1.0 Universal Public Domain Dedication; credits: *P. vitulina*: Tracy Heath, 29 June 2013; *Z. californianus*: Margot Michaud, 4 April 2021; *U. maritimus*: Margot Michaud, 4 April 2021; *T. truncatus*: Steven Traver, 22 Feb 2012).

Lifespan equality and life expectancy increased gradually between the earliest and latest periods for both sexes of the four species (figure 2; electronic supplementary material, figures S5–S7). Between the first available baseline period and the most recent period (2005–2020), they all showed an increase in life expectancy ranging from 1.04 to 3.40 times (electronic supplementary material, table S1). Both sexes of all species experienced a steep increase in life expectancy and lifespan equality between 1990–2004 and 2005–2020, as shown by the KL discrepancies which, for the most part, were close to 1, indicating no overlap between the posterior densities (electronic supplementary material, table S3). The exception was males of common bottlenose dolphins for which the lifespan equality increased between the two first periods studied (1947–1989 and 1990–2004) and remained similar in the most recent period (K-L = 0.1, electronic supplementary material, table S3). Moreover, females of the four species generally had similar life expectancy and lifespan equality to males during the first periods (K-L values between 0.04 and 0.22), while, for the last period, females had longer life expectancy and higher lifespan equality than males, particularly for the California sea lion and polar bear (electronic supplementary material, figure S8 and table S4).

Overall, for both sexes of all four species, life expectancy and lifespan equality populations in zoological institutions were higher than for their wild counterparts, particularly during the two latest periods (figure 2; electronic supplementary material, table S1). In most cases, the wild values fell on the lower end of the posterior distributions of life expectancy and lifespan equality of the populations in zoological institutions, and for the most part with quantiles less than 0.001, indicating they were much lower than the zoo-held estimates (electronic supplementary material, table S5). Indeed, the life expectancies of females living in zoological institutions of the four species currently are 1.65–3.55 times longer than their wild counterparts, and for males, life expectancies in zoological institutions are 1.77–3.24 times longer than in the wild. Life expectancies for both sexes for the wild are broadly comparable to those obtained for pre-1990 zoos, except for the polar bear, where survival in zoological institutions has been consistently higher. Finally, lifespan equality values for populations in zoological institutions in the latest period were higher than the values obtained for the wild populations, while the wild values, in general, were much lower (less than 0.001 percentile) than the mean in the latest period. Notable exceptions were one population of harbour seal and both populations of bottlenose dolphin, for which the wild values fell below the lower 33 percentile, suggesting no difference in lifespan equality.

## 4. Discussion

**Figure 3.**
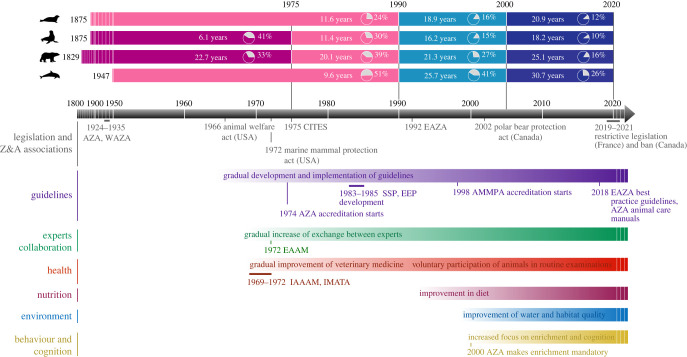
Summary of long-term changes in female life expectancy and potential contributing factors for zoo-held harbour seal, California sea lion, polar bear and common bottlenose dolphin. Female life expectancy was derived from 1 year of age, and first-year mortality is shown in inset pie charts. See electronic supplementary material, table S6 for references and more details. AMMPA, Alliance Marine Mammal Parks and Aquariums; AZA, (American) Association of Zoos and Aquariums; CITES, Convention on International Trade in Endangered Species of Wild Fauna and Flora; EAAM, European Association for Aquatic Mammals; EAZA, European Association of Zoos and Aquaria; EEP, EAZA Ex-situ Programme; IAAAM, International Association for Aquatic Animal Medicine; IMATA, International Marine Animal Trainer's Association; SSP, Species Survival Plan; WAZA, World Association of Zoos and Aquariums; Z&A, zoos and aquariums. Silhouette images extracted from Phylopic.org (License CC0 1.0 Universal Public Domain Dedication; credits: *P. vitulina*: Tracy Heath, 29 June 2013; *Z. californianus*: Margot Michaud, 4 April 2021; *U. maritimus*: Margot Michaud, 4 April 2021; *T. truncatus*: Steven Traver, 22 Feb 2012).

We analysed changes in survival and longevity across time for four species of marine mammals living in zoological institutions, and found a general decrease in first-year mortality and an increase in life expectancy and lifespan equality. These results show that improvements in population welfare are ongoing. However, the timing and extent of changes varied among species, potentially reflecting differences in the implementation and effectiveness of welfare-promoting measures per species (figure 3). This increase in life expectancy and lifespan equality results from a delay of early deaths and the subsequent concentration of deaths at older ages, while a reduction in first-year mortality did not solely cause it. Indeed, similar results were obtained when these demographic metrics were estimated from birth and from age of sexual maturity. Such associated increases in life expectancy and lifespan equality have also been observed in comparisons of pre-industrial and industrial human societies [9] and in comparisons of wild and zoological institutions populations of non-human primate species [12].

Our results support reported improvements in life expectancy and first-year survival for the common bottlenose dolphin and California sea lion in US facilities [4,5,38]. This improvement was also observed for orcas [8], a species closely related to the common bottlenose dolphin, although the results are debated [7,39,40]. However, to our knowledge, this is the first report of longevity improvements for harbour seal and polar bear in zoological institutions. Among humans, increases in life expectancy and lifespan equality have been attributed to the effect of social, economic and public health advances on mortality rates [9]. Similarly, animals living in modern zoological institutions are shielded from many pressures affecting mortality (e.g. starvation, disease, parasites, environmental impacts) [41]. Interestingly, at the population level, the demographic patterns of marine mammals in zoological institutions across time (and between wild and zoo populations) are qualitatively similar to those observed during the industrial revolution in humans [9]. Specific changes in zoological management practices over the last decades (figure 3; electronic supplementary material, table S6) likely have contributed to the demographic improvements we found. In the nineteenth century, many zoological institutions started as menageries, where conditions for animals were poor, and survival was low [41]. In the 1960s and 1970s, practical experience increased, and laws were passed to improve species conservation in the wild and animal care in zoological institutions (e.g. the Animal Welfare Act and the Marine Mammal Protection Act in the USA). In the 1970s and 1980s, the establishment of regional zoo associations, accreditation standards [42], coordinated breeding programmes, shared databases [13], and professional networks further enabled zoological institutions to acquire and share knowledge about their animals and collectively improve welfare standards.

The most significant improvements in demographic metrics for the four marine mammal species occurred in the 1990s onwards, potentially linked to the implementation of advanced veterinary [43,44], environmental [45], nutritional [46,47] and enrichment [48] measures (figure 3). Importantly, the voluntary cooperation of animals in routine examinations, achieved through training and positive reinforcement [49], has reduced the need for anaesthesia and facilitated regular health monitoring. Increased knowledge of species-specific needs has underpinned environmental enhancements, including advances in water treatment, habitat design and enrichment [45]. Animal diets in modern zoological institutions typically account for the nutritional requirements of different species, sexes and life stages [46,47], and often use human-grade food, vitamin supplementation, and improved provision through training and enrichment. Advances in animal welfare science have also shown that cognitive enrichment, training and species-specific social management are more essential components of cetacean welfare than habitat size [49]. As a result, enrichment is mandatory in zoos accredited by most regional associations of zoos and aquariums (e.g. AZA [42], EAAM [50]).

Our findings also highlight that populations of the four species living in zoological institutions show, in the most recent period, a longer life expectancy than their wild counterparts. Our results confirm previous findings on these species [4,5]. However, comparisons with wild populations must be made cautiously (e.g. see [4]). For example, it is important to consider that anthropogenic threats in natural environments affect longevity measures [51]. For instance, the bottlenose dolphin population of the Indian River Lagoon system [16] is known for its health problems caused by anthropogenic activities [52,53]. Nevertheless, we obtained comparable results using another population living in the Mississippi sound region in the Gulf of Mexico [20]. Importantly, research on primates has shown that life expectancy and lifespan equality of different populations of the same species fall within a linear continuum, whereby populations under poor conditions (e.g. high anthropogenic pressure) fall at the lower end of the continuum, and those under protected conditions (e.g. populations in zoological institutions) at the upper end [12]. Notably, this continuum is not an artefact of how these two metrics are calculated, but it is driven by biological constraints inherent to each species. Our results support that different populations of these four species of marine mammals recapitulate their own linear continuum, with the populations in zoological institutions in the recent period falling at the upper end. With the increase of anthropogenic pressure on natural habitats coupled with climate change, we can expect deteriorating conditions for wild populations resulting in changes along this gradient in the future.

It is important to note that for humans and other animals, long life may not necessarily equate to quality of life [54,55]. However, the significant increase in both measures (i.e. life expectancy and lifespan equality) likely reflects certain factors that may improve quality of life (figure 3), as seen in humans [9]. For example, higher life expectancy positively correlates with the number of years a human lived free of ill-health and disability [54]. Yet, our study does not assess individual-level welfare or quality of life, which is essential to advance animal care and develop a holistic understanding of animal welfare. Unfortunately, the development of large-scale studies evaluating individual-level welfare has been hampered due to the lack of standardized assessment protocols across individuals, species and space (see [3,6]). Future research should focus on designing scientifically sound quality-of-life indicators based on globally standardized data (e.g. from ZIMS).

As for ageing human societies [54], increases in life expectancy and the number of individuals reaching old age have important implications for animal care and population management in zoological institutions. Our survival analysis, together with reproduction models, could support collection planning by zoos, for example to balance the allocation of habitable space between geriatric and young individuals while considering population management goals [56]. In both wild mammals [57] and humans [9,58], females tend to exhibit higher life expectancy and lifespan equality than males. We found not only a female life expectancy and lifespan equality advantage in zoological institutions, but also an increasing difference over time, suggesting that this bias may be amplified with improved management practices. Our results may also reflect differences in husbandry needs or practices between males and females, which should be further investigated to inform sex-specific animal management.

Before 1975, only 3–48% of individuals of the four species were born in zoological institutions, compared to 82–92% in the current period (electronic supplementary material, figure S4). This change likely reflects the effect of the implementation of the Convention on International Trade in Endangered Species of Wild Fauna and Flora (CITES) as well as national conservation strategies such as the US Marine Mammal Protection Act. We did not detect an effect of the provenance on the mortality of marine mammals living in zoological institutions, except for the common bottlenose dolphin. Our findings do not agree with Small & DeMaster [5] that found significantly higher annual survival for captive-born California sea lions compared to wild-born in US facilities, and with no differences in survival for the provenance of common bottlenose dolphins. Due to a negligible effect on the overall mortality pattern of the three other species, we did not include the provenance in the main analysis for the four species. Therefore, we acknowledge that, for the common bottlenose dolphin, the positive influence of the improvement of environment of life in zoological institutions (figure 3) can be, at least partially, due to the decrease of the number of wild-caught individuals living in zoological institutions between the first period (before 1990) and the two more recent periods. Indeed, the lifespan equality of males significantly improved between the two first periods but not in the recent one. The improvement on both metrics across the three periods observed for the females but not for males insinuates between-sexes differences in the needs in captivity for this species.

Banning species from modern zoological institutions without strong welfare evidence may represent missed opportunities to (i) acquire species-specific knowledge to support conservation efforts [59–62], (ii) care for confiscated animals [63] or serve as a temporary home for rescued ones, (iii) maintain assurance populations that help preserve species and their genetic diversity [64] until threats in the wild are abated [65], to allow potential reintroduction into the wild [60], and (iv) promoting public engagement and behaviour change through education. Therefore, if countries wish to legislate on housing marine mammals in zoological institutions, it is essential that they consider the potential of these animals for research, education and conservation, in addition to any evidence of compromised welfare. For instance, the governments of France (2021) and Spain (2023) passed bans on cetaceans for shows (figure 3) but, based on our preliminary results, the bans did not extend to individuals involved in research projects with conservation and welfare goals. Importantly, *ex situ* conservation programmes can play a major role in preventing species extinction for species severely threatened in the wild [60]. Although to date no marine mammals have been saved from extinction through *ex situ* programmes (e.g. unsuccessful attempts to recover the population of vaquita, *Phocoena sinus* [66] but see the recent survey [67]; see [68] for promising development for the Yangtze finless porpoise (*Neophocaena asiaeorientalis asiaeorientalis*) in China), the potential of *ex situ* conservation is still recognized by leading international conservation institutions. For instance, the ‘Ex Situ Options for Cetacean Conservation’ report of the International Union for Conservation of Nature (IUCN), stresses the importance of *ex situ* research and management for the sustainable conservation of highly threatened dolphins [62].

While the international non-governmental organization Species360 hosts ZIMS (the world's largest zoological database, with more than 10 million records for 22 000 species), it does not include information from all zoological institutions. As such, ZIMS data and our analysis may not capture all the variation expected in diverse care practices and demographic parameters for the four species. For instance, ZIMS does not include data from some institutions with extensive marine mammal expertise and with a proven track record of following high standards in care (e.g. the Dolphin Company, SeaWorld), nor from some non-accredited zoos which may house animals under lower standards of care. Further analyses incorporating additional datasets could improve our understanding of the observed trends, especially for the bottlenose dolphin, the only obligate aquatic species in our study which also had the smallest sample size.

## 5. Conclusion

Our findings highlight the effects of improvements in species-specific knowledge, management and care practices in modern zoological institutions, as the four species analysed today live on average 1.65–3.55 times longer than their wild counterparts. In addition, we found a significant increase in first-year and adult survival across time in marine mammal facilities. Thus, our results contradict arguments of poor or lower survival in zoological institutions than in natural habitats. We acknowledge that there are other perspectives regarding keeping marine mammals in zoos, such as different ethical viewpoints and differences in husbandry and welfare conditions across institutions. Further studies on individual welfare on a global scale will be important to assess which practices have driven these improvements in survival. Therefore, science-driven species management, welfare and conservation programmes will ensure a better understanding of species biology and needs, that will maximize our chances of preventing a species' functional extinction.

## Data Availability

Data for animals in zoos and aquariums are available from Species360 through a reasonable research request (https://conservation.species360.org/data-sharing/). Aggregated and anonymized data to reproduce the analyses are available in electronic supplementary material, appendix S1 [69].
